# Case of Hydrops Amnii

**Published:** 1862-07

**Authors:** A. E. Johnson

**Affiliations:** St. Anthony, Minnesota


### CASE OF HYDROPS AMNII.

By A. E. JOHNSON, M. D., of St. Anthony, Minnesota.

May 15th, 1862, I was called to attend Mrs. L., a lady about thirty-five years old, pregnant with her sixth child. She had arrived at full term of her pregnancy. She had suffered severely with headache, and also extreme dyspnœa for the last two months, which became intolerable on assuming the recumbent posture for more than a few moments at a time. She was also harrassed with violent cough for six weeks prior to her confinement. The abdominal walls were distended to their utmost, so much so that she suspected a triple or quadruple birth. The examination per vaginam detected the os well dilated. The pains had been slight and irregular for eighteen hours. The presentation could not be discovered. The membranes felt rough and very tense. A second examination, at the end of two hours, discovered no advance. The contractions had slightly increased in force and frequency, though still irregular. On placing my left hand upon the abdomen over the fundus of the uterus, and making pretty smart indentations upon the strong firm membranes with the index finger, the child was distinctly felt by the left as it impinged against the walls of the uterus and abdomen, pressing hard against the ziphoid cartilage of the sternum. I at once concluded that there was a great excess of liquor amnii, above the normal amount. I at once proceeded to rupture the membranes, and after several unsuccessful attempts with the finger I succeeded by the use of a strong knitting needle, there being no other instrument at hand, after considerable exertion.

The escape of an almost incredible amount of liquor amnii gave great relief to my patient, and to which the before enormously distended and apparently half paralyzed uterus soon responded with energy sufficient to deliver a very small well formed, with one exception, feeble female child by the breech presentation. With much exertion the child was resuscitated. The time occupied in its delivery after the rupture of the membranes did not exceed forty-five minutes. It lived but twenty-four hours. There was an abnormal growth upon its right cheek about the size of an ordinary nipple.

After waiting a proper time the placenta had to be extracted, one-half of which was above an almost irresistible hour-glass contraction. The membranes were extremely thick, and presented upon their fœtal surface the evidence of many inflammatory patches. The placenta was small.

The exact amount of liquor amnii could not be estimated. The patient was laying upon a thick feather bed and a straw tick, well filled beneath it. Over this was a quilted cotton comfortable folded in four thicknesses, and the patient's quilted skirt loosely around her person. The amount of water which escaped was sufficient to saturate all of these and two full gallons passed through, which was caught in a pail beneath the bed. The whole amount, I think, could not have been much short of four gallons.

I had attended this lady four times in child birth prior to this, and nothing unusual had occurred.

Query: What was the pathological cause of this enormous accumulation?

Here was no constitutional peculiarity or syphilitic taint, which Churchill and Burns think "may be amongst the more remote causes of this disease." In this case, its cause must have been an inflammation of the amniotic membrane, the evidence of which was well marked.

M. Mercier, M. Davillier, M. Desmarais, M. Chailly, and others, speak of inflammation of the amniotic membrane as the most frequent cause of dropsy of the amnios.

Drs. Burns, Remsen, Kyll, and others, think "it may be connected with diseases of the placenta, such as cysts, tubercles, induration, dropsy, &c." But in this case, the placenta was to all appearance healthy and natural.

